# Takayasu's Arteritis in Pregnancy: A Rare Case Report from a Tertiary Care Infirmary in India

**DOI:** 10.1155/2017/2403451

**Published:** 2017-02-07

**Authors:** Sheeba Marwah, Monika Rajput, Ritin Mohindra, Harsha S. Gaikwad, Manjula Sharma, Sonam R. Topden

**Affiliations:** ^1^Department of Obstetrics and Gynecology, VMMC and Safdarjung Hospital, New Delhi 110029, India; ^2^Department of Internal Medicine, VMMC and Safdarjung Hospital, New Delhi 110029, India

## Abstract

*Background*. Takayasu's arteritis (TA) is a rare, chronic, inflammatory, progressive, idiopathic arteriopathy, afflicting young women of reproductive age group, causing narrowing, occlusion, and aneurysms of systemic and pulmonary arteries, especially the aorta and its branches. During pregnancy, such patients warrant special attention. An interdisciplinary collaboration of obstetricians, cardiologists, and neurologists is necessary to improve maternal and fetal prognosis. Here a case is reported where a patient with diagnosis of TA, complicated by neurological sequelae, successfully fought the vagaries of the condition twice to deliver uneventfully.* Case*. 25-year-old G2P1L1 presented at 34 weeks of gestation, with chronic hypertension, with TA, with epilepsy, and with late-onset severe IUGR. Following a multidisciplinary approach, she delivered an alive born low birth weight baby (following induction). Her postpartum course remained uneventful.* Conclusion*. Pregnancy with TA poses a stringent challenge to an obstetrician. Despite advancements in cardiovascular management and advent of new-fangled drugs, the optimal management for pregnant patients with this disease still remains elusive.

## 1. Introduction

Takayasu's arteritis (TA), also known as pulseless disease/aortoarteritis/“young female arteritis,” is a rare chronic inflammatory progressive large vessel vasculitis (LVV) of unknown etiology afflicting women of childbearing age [1–4]. It was first described by the Japanese ophthalmologists Mikito Takayasu and Onishi [5]. Its incidence is reported to be 13 cases per million population [1]. It is predominantly seen in the women of Asian origin [6]. It leads to narrowing, occlusion, and aneurysms of systemic and pulmonary arteries in the body, affecting primarily the aorta and its branches [3, 7].

Pregnancy as such has no effect on the evolution of the disease;however, its peak incidence is in second and third trimesters. Thus, such patients warrant special attention during the peripartum period owing to the likelihood of development of complications such as hypertension, multiple organ dysfunction, and stenosis hindering regional blood flow leading to restricted intrauterine fetal growth and low birth weight in babies [8–10]. Delay in diagnosis is quite common, so patients often conceive without prior knowledge of having TA, or having initiated specific treatment against it [2]. Ideal management for pregnant patients with this disease still poses a stringent challenge, especially in the light of movement towards multicentric LVV research across the world, coupled with the recent availability of levitating pool of targeted drugs. An interdisciplinary collaboration of obstetricians, cardiologists, rheumatologists, and neurologists is often necessitated for an optimal maternal and fetal prognosis. Taking into consideration the small-scale researches in literature so far on active TA in pregnancy, especially in LMIC countries, here a case is described and literature reviewed to enlighten the obstetricians on fetomaternal outcome and management of this infrequent, but not uncommon clinical entity encountered nowadays.

## 2. Case Report

A 25-year-old primigravida was admitted in the hospital as pregnancy with chronic hypertension (not on any antihypertensives), at 36 completed weeks in active labor. She was a known case of active TA on treatment (taking prednisolone 2 mg, aspirin 150 mg, and clopidogrel 75 mg OD.) She was booked and supervised throughout her pregnancy at the same hospital, in liaison with cardiologists. Her past and personal histories were thoroughly reviewed in outpatient department; she had been a bidi smoker since teenage, one-two per day. She had surgical correction of complete stenosis of right common carotid artery (CCA) and right vertebral and subclavian artery by percutaneous stenting of right brachiocephalic (size 7 × 39 mm) and right CCA (size 9 × 30 mm) in some peripheral hospital. She had no history of other comorbidities like IBD and sinusitis. She had an uneventful antenatal and intrapartum period and delivered a healthy neonate weighing 2.8 kg. During her postpartum period, all necessary investigations were done and consultations were taken, and she was discharged on drugs after one week in stable condition, with the advice for regular follow-up and abstinence from smoking. During her subsequent visits, her MRA scan showed markedly attenuated flow in left CCA, for which she was conservatively managed by cardiologist.

**Figure 1:**
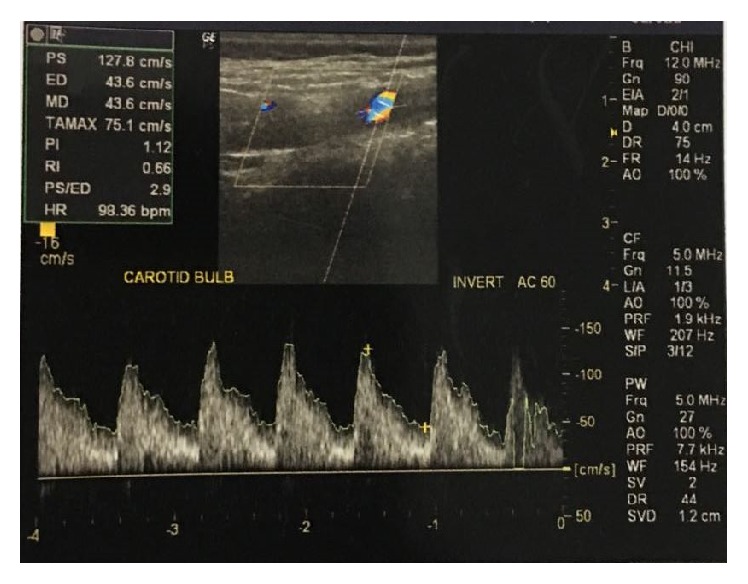
Carotid artery Doppler of the patient.

**Figure 2:**
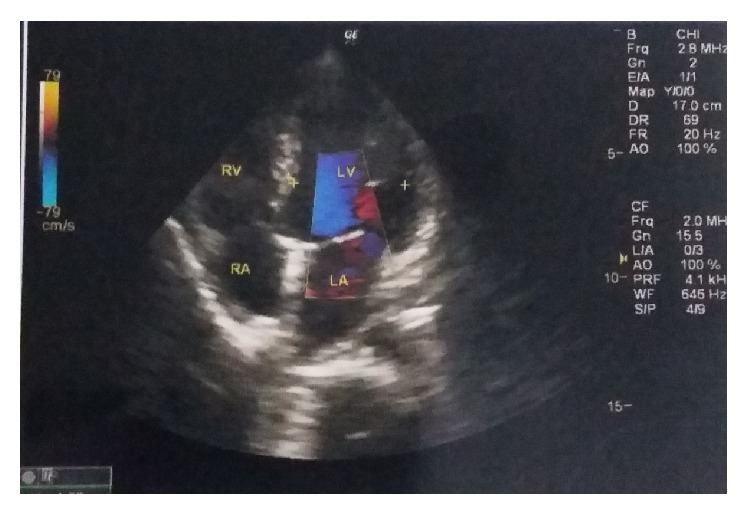
Echocardiography of the patient.

**Figure 3:**
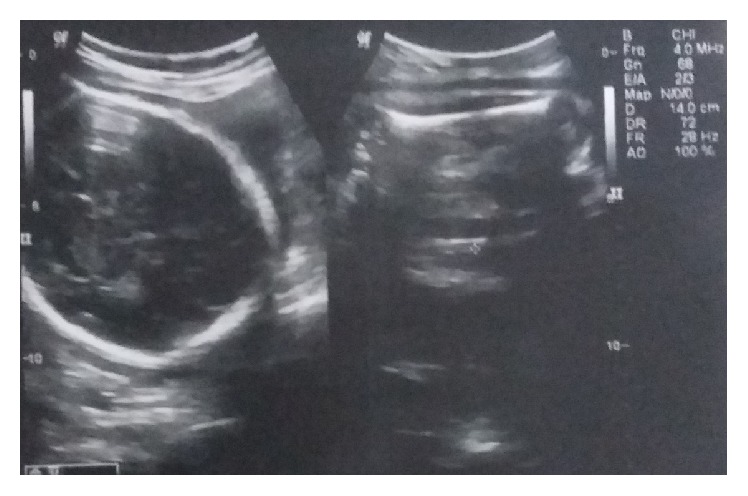
Obstetric ultrasound of the patient.

One year later, she had an accidental second conception but could not get herself booked for antenatal care anywhere till the third trimester, due to social and financial issues. At her first visit at 34 weeks in the institute, she was admitted for safe confinement for severe fetal growth restriction and neurological sequelae of aortoarteritis in the form of tonic-clonic convulsions (three episodes in last 24 hours before admission). She gave history of myalgias, arthralgias, and fever off and on throughout her pregnancy, despite continued glucorticoid treatment. On admission, her BP was 200/110 in ankle and pulse was 100/min. There was no albuminuria. Per-abdomen examination showed a fundal height corresponding to 26 weeks with faintly audible fetal heart sound. She was put on strict fetomaternal surveillance. After thorough history taking, cardiology, and CTVS references were taken, her anticoagulants were stopped in v/o anticipated termination of pregnancy, and she was started on labetalol 300 mg in divided doses and continuous BP monitoring (noninvasive). A neurology consultation was done, and levetiracetam 1000 mg was started to take care of seizures. All routine antenatal and specific blood investigations (INR, PT, and APTT) were normal. Though CRP was also normal, it was on a higher side of normal range (3 mg/dL). ESR was found to be raised being equal to 33 mm/1st hour. Echocardiography was performed which revealed mild concentric LVH, moderate AR (38 mm diameter), mildly thickened aortic valve leaflets, and ejection fraction 63% (Figure 2). Carotid and vertebral Doppler reported mild stenosis of poststent segment, with 65–70% stenosis of left CCA and ICA, which was suggestive of progressive active TA in the patient (Figure 1). A renal Doppler was also done that was normal. Her obstetric ultrasound with Doppler velocimetry showed a single live growth restricted fetus with oligohydramnios (AFI-5CMS) and severely deranged diastolic flow in umbilical artery (Figure 3). The couple were counselled adequately about fetomaternal prognosis and after their informed consent, she was given two doses of betamethasone for lung maturity, following which induction of labor was done with cervigel at 35 weeks two days. Her labor progressed well and she delivered live boy baby weighing 1.2 kgs, who was transferred to nursery, being very low birth weight baby, and discharged after one month. Patient's intrapartum and postpartum period were uneventful and she was discharged on higher dose of prednisolone, amlodac 7.5 mg, and levetiracetam. At the time of this writing, she is convalescing well, with both mother and baby doing fine, and following up periodically with cardiologists, rheumatologists, and gynecologists.

## 3. Discussion

Takayasu's arteritis is a LVV with aortic inflammation leading to proximal occlusion and/or aneurysms of carotid, subclavian, pulmonary, iliac, and renal arteries [3, 18]. Mean age is typically reported between second and third decade of life [19–21]. Its etiology remains primarily idiopathic. Autoimmunity, sex hormones, and genetic (predisposition of the human leukocyte antigen, HLA BW52) factors have often been hypothesized as plausible factors causing it [22]. These factors were conspicuously absent in the case described.

Various types of TA have been acknowledged in the past: type I (disease embroiling aortic arch and its branches), type II (lesions constrained to descending thoracic aorta and abdominal aorta), type III (patients with characteristics of types I and II), type IV (involvement of pulmonary artery), and type V (combined features of types IIb and IV) [10]. The above described patient was labeled as type II TA. The disease can also be classified into stages as per the presence of major complications such as hypertension, retinopathy, aneurysms, and aortic insufficiency [23]: stage I (no complications observed), stage IIa (patients having only one of these complications), stage IIb (patients with only one of these complications, but the severe form), and stage III (more than one complication is present). The patient presented here was in stage II, but during pregnancy her hypertension was compensated and her aneurysm had been corrected.

Pregnancy does not interfere with disease progression [1–3]; but TA has several adverse implications on pregnancy like abortions, preeclampsia, IUGR, IUD, and abruption [21, 24, 25], as seen in our case in the form of late-onset IUGR. Etiology of IUGR may be impaired placental blood flow. Incidence of IUGR is high when bilateral renal involvement is present [3]. Gatto et al. reported fetal growth restriction in 51.7% of fetuses in a study in India [21]. More than 60% of patients have some kind of complications and the four most important ones are Takayasu's retinopathy, secondary hypertension, aortic regurgitation, and aneurysm formation. Hypertension is fairly common due to reduction in elasticity and narrowing of the arteries, besides abnormalities in functioning of aortic and carotid baroreceptors function [26]; it should be prudently contained during pregnancy, as severe renovascular hypertension, cardiac involvement, or pulmonary hypertension is associated with poor fetomaternal prognosis like abortion, preterm labor, and low birth weight babies [2]. Blood pressure in such patients should be also measured in the lower extremity to pick up blood pressure discrepancies; like in our case BP recordings in lower extremity were higher than upper extremity. Besides, pulselessness of unilateral or bilateral radial arteries and vascular bruit should be seen in all cases of hypertension. Involvement of abdominal aorta is associated with adverse pregnancy outcomes, which was fortunately absent in the present case. Arterial ultrasound Doppler, quantifying the flow in the uterine arteries, is beneficial in evaluation of fetal well-being and growth in women with TA.

Diagnosis is usually based on clinical manifestations, inflammatory markers (acute phase reactants), and arteriography demonstrating aortic stenosis and of its branches. Common features of active TA are fatigue, myalgia, arthralgia, and low-grade fever in initial stages and intermittent claudication, visual defects, and fainting attacks in later stages. Many may be diagnosed after clinical examination, when one or more peripheral pulses are not palpable or blood pressures vary in two limbs. However, computed tomography or magnetic resonance angiography can detect TA even before the development of severe vascular compromise as in our case [27]. Recently, 18 FDG-PET scan has been added as an adjunct imaging modality in the armamentarium of rheumatologists and cardiologists to diagnose LVV, with a pooled sensitivity and specificity of 70.1% and 77.2%, respectively [28]. But this is currently not available in our hospital. However, the gold standard for diagnosis still remains as vessel biopsy [27] which could not be performed in our case. Presence of constitutional symptoms (postsurgical treatment and glucocorticoids), progressive involvement of other branches of aortic arch, raised ESR, and onset of seizures in the patient clinched the diagnosis of active TA in her. Contribution from other specialties is fundamental for detection and treatment of disease complications. In the case presented here the disease was not compensated with drug therapy and lesions in the aortic arch and aortic valve had been partially corrected; but the patient did not have a recent arteriography or angio-MRI of large vessels due to loss to follow-up.

Involvement of large vessels in our case excludes granulomatosis with polyangiitis and Behcet's disease. Another cause of LVV is giant cell arteritis, which was one of the primary differential diagnoses in our case. But younger age, lower values of raised ESR, absence of any new onset headache, visual symptoms, and jaw claudication symptoms favored the diagnosis of TA [29]. Fibromuscular dysplasia is ruled out by the presence of raised ESR in our patient [29]. IgG4 related vasculitis was eliminated by the absence of lymphadenopathy, normal upper abdomen scan, and unresponsiveness of the patient to prednisolone.

Management of TA entails an interdisciplinary approach with involvement of obstetricans, anesthesiologists, cardiologists, rheumatologists, and neonatologist in a tertiary care center. The aims are control of inflammation, prevention and treatment of complications like hypertension and revascularization by percutaneous angioplasty, use of endoprosthesis, or surgical correction for occlusive and stenotic lesions.

Preconception counselling is essential regarding dosage adjustment or cessation of cytotoxic drugs, folic acid supplementation in the periconceptional period, and optimal timing of pregnancy. Pregnancy should be ideally planned in remission phase. There should be an early booking with regular antenatal supervision. Along with routine antenatal visits, serial monitoring of blood pressure, renal function, cardiac status, and preeclamptic screening are vital in such patients. Fetal surveillance including daily fetal kick count, gravidogram, serial fetal biometry, biophysical profile, and fetal Doppler is also imperative as per requirements [30].

Blood pressure monitoring can be challenging in patients with pulseless peripheral arteries. In most cases described in literature, and in the present case, it was possible to use the noninvasive technique [31]. If there is a large difference in blood pressure in upper and lower limbs, one must encourage recording it in both limbs. To evaluate limb perfusion a good alternative is to assess blood pressure in one limb and oximetry in the other; the same was done in the extant case. Antihypertensive drugs and antiplatelets can be started as per need, as was in the present case. TA may respond symptomatically to corticosteroid therapy (first line drugs) at a dose of 1-2 mg/kg/bodyweight for 4 weeks followed by slow tapering. However, chronic use of corticosteroids could lead to suppression of adrenal gland activity with inadequate release of endogenous corticosteroids in moments of stress, such as surgeries [32]. Also immune-suppressors including methotrexate and azathioprine are used.

Utilization of immunomodulatory agents like mycophenolate mofetil, infliximab, tocilizumab, leflunomide, and abatacept has gained momentum in recent times for treatment of TA, especially in refractory cases [33, 34]; however their safety in pregnancy has not yet been established. Hence these are generally avoided in pregnancy or used after a meticulous assessment of the risk/benefit ratio for the patient [35, 36].

Vaginal delivery is the preferred mode, and epidural analgesia has been advocated for labor and delivery. In women with hypertension, delivery should be abbreviated by the use of outlet forceps. In women with stages IIb and III, LSCS is preferred to prevent cardiac decompensation due to increased blood volume and blood pressure observed during uterine contractions and increased cardiac output observed during labor. Our patient was hemodynamically stable and was also induced as she belonged to group IIa and she did not have additional supplementary investigation of the abdominal vessels.

Patients with metallic valvular prosthesis should be maintained anticoagulated during pregnancy. The choice of medication should take into account the probable due date and reversibility of the method. Heparin should be discontinued 4 to 6 hours before anesthesia, and it can be reversed with protamine if the gravida goes into labor or in case of bleeding. Patients on prophylactic doses of enoxaparin should receive their last dose 12 hours before anesthesia. In the case of therapeutic doses the drug should be discontinued 24 hours before anesthesia.

Table 1 shows the clinical presentation, maternal complications, mode of delivery, and fetal/neonatal outcome of recently reported cases, in comparison with the reported case [11–17, 26].

The overall five-year survival rate after diagnosis was 83.1%. Death typically is a consequence of congestive heart failure or cerebrovascular events. The survival is better in patients without a progressive course and in those below 35 years of age. Early diagnosis with proper medical or surgical management is essential for a good prognosis. A high index of clinical suspicion in patients presenting with pulseless peripheral vessels could be kept in mind to optimize the management following a multidisciplinary approach.

## 4. Conclusion

Pregnancy with TA presents as an onerous medical condition to manage for an obstetrician.

## Figures and Tables

**Table 1:** 

Study	Parity	Clinical presentation	Maternal complications	Mode of delivery	Fetal outcome
Leal et al. (2011) [11]	G4P3l3	Precordial squeezing pain	Controlled HTN	Elective LSCS at 39 weeks	2.7 kgs live baby

Rengaraj and Rani (2015) [12]	PGR	Dyspnea PROM	GTCS	Outlet forceps	2.4 kgs live baby

Nalini and Santa (2015) [13]	PGR	Oliguria	IUGR Uncontrolled hypertension	Elective LSCS at 37 weeks	2.4 kgs live baby

De Lucena et al. (2008) [14]	G3P1L0	Dyspnea Palpitations Intermittent claudication	Uncontrolled HTN	Elective LSCS at 37 weeks	2.1 kgs

Soma-Pillay et al. (2015) [15]	G2P1L1	Claudication in LL	Compensated HTN	Elective LSCS	2.3 kgs

Satia et al. (2016) [16]	G3A2	Exertional dyspnea Palpitations dizziness	Dilated cardiomyopathy	MTP at 6 weeks	

Khandelwal and Gandhi (2016) [17]	G7P1A5	Blurring of vision	Grade IV hypertensive retinopathy	Elective LSCS at 37 weeks	2.5 kgs live baby

Present report (2017)	G2P1L1	Pain in right lower limb	Controlled HTN Neurological Sequelae IUGR	NVD	1.2 kgs live baby
